# Nitazoxanide mitigates methotrexate hepatotoxicity in rats: role in inhibiting apoptosis and regulating endoplasmic reticulum stress

**DOI:** 10.3389/fphar.2024.1491249

**Published:** 2024-12-02

**Authors:** Nevertyty Mohamed Mahmoud, Shimaa M. Elshazly, Fatma El-shaarawy, Sawsan A. Zaitone, Afaf A. Aldahish, Gehan A. Ahmed, Manal S. Fawzy, Sheka Yagub Aloyouni, Sally Y. Abed, Tahani Saeedi, Shaimaa S. El-Sayed

**Affiliations:** ^1^ Department of Clinical Pharmacology, Faculty of Medicine, Zagazig University, Zagazig, Egypt; ^2^ Department of Pharmacology and Toxicology, Faculty of Pharmacy, Zagazig University, Zagazig, Egypt; ^3^ Department of Biochemistry, Faculty of Pharmacy, Sinai University, Arish, Egypt; ^4^ Department of Pharmacology and Toxicology, Faculty of Pharmacy, Suez Canal University, Ismailia, Egypt; ^5^ Department of Pharmacology and Toxicology, Faculty of Pharmacy, University of Tabuk, Tabuk, Saudi Arabia; ^6^ Department of Pharmacology, College of Pharmacy, King Khalid University, Abha, Saudi Arabia; ^7^ Forensic Medicine and Clinical Toxicology Department, Faculty of Medicine, Zagazig University, Zagazig, Egypt; ^8^ Department of Biochemistry, Faculty of Medicine, Northern Border University, Arar, Saudi Arabia; ^9^ Center for Health Research, Northern Border University, Arar, Saudi Arabia; ^10^ Research Department, Natural and Health Sciences Research Center, Princess Nourah bint Abdulrahman University, Riyadh, Saudi Arabia; ^11^ Department of Respiratory Care, College of Applied Medical Science in Jubail, Imam Abdulrahman Bin Faisal University, Jubail, Saudi Arabia; ^12^ Department of Pharmacology and Toxicology, School of Pharmacy, Taibah University, Medina, Saudi Arabia

**Keywords:** nitazoxanide, methotrexate, hepatotoxicity, endoplasmic reticulum stress, rat

## Abstract

**Objectives:**

Hepatotoxicity is a severe outcome of methotrexate (MTX) therapy, limiting its clinical use and contributing to its related morbidity and mortality. This study investigated the hepatoprotective effects of nitazoxanide (NTZ), an antiprotozoal drug, against MTX-induced hepatotoxicity and whether endoplasmic reticulum (ER) stress-modulation underlies the expected beneficial effects of NTZ.

**Methods:**

Thirty-six rats were allocated to six groups, one control group and five MTX groups, where induction of hepatotoxicity was achieved via injecting MTX (20 mg/kg). Groups were assigned as MTX-vehicle, NTZ-100, and NTZ-200 groups (at 100 and 200 mg/kg/day, gavage, respectively), N-acetyl cysteine (NAC) group (500 mg/kg), and 4-phenyl butyric acid (4-PBA) group (150 mg/kg, i.p). Liver function enzymes in serum, hepatic oxidative stress, proinflammatory cytokines, apoptosis, and ER-stress biomarkers were assessed. A histopathological examination was performed.

**Results:**

Treatment with NTZ lessened the serum liver enzymes, reduced malondialdehyde (lipid peroxidation product), enhanced antioxidant capacity, attenuated proinflammatory cytokines, and suppressed apoptosis. The protective effect of NTZ was dose-dependent, and the findings observed with the high-dose NTZ were similar to those obtained with the ER-stress inhibitor (4-PBA).

**Conclusion:**

NTZ exerted a hepatoprotective effect in MTX-challenged rats that is mediated via modulation of ER stress and inhibiting apoptosis.

## Graphical Abstract

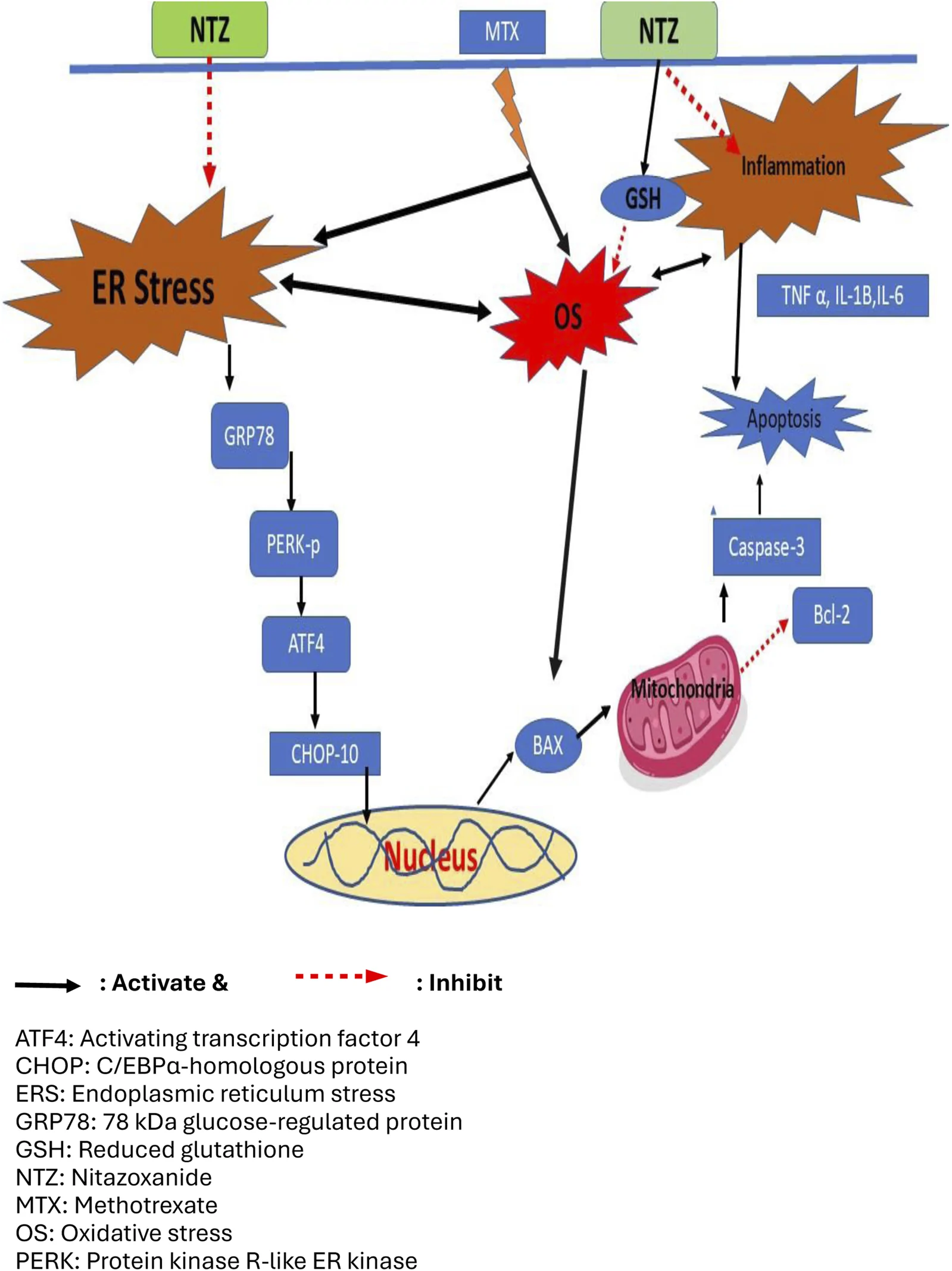

## 1 Introduction

Methotrexate (MTX) is a dihydrofolate reductase inhibitor and chemotherapeutic agent with proven efficiency against cancers and rheumatoid arthritis (Kozminski et al., 2020). Despite the high efficacy-to-toxicity ratio, MTX toxicity remains a significant concern that leads to a limitation on its use (Shea et al., 2013). Adverse outcomes among patients on MTX include hepatotoxicity, hematotoxicity, testiculopathy, and lung problems (Shea et al., 2013). MTX-induced liver toxicity is characterized by increased levels of aminotransferases, the development of hepatic steatosis, and progression to liver fibrosis and cirrhosis. This condition may be linked to the depletion of folate reserves in the liver due to MTX treatment (Conway and Carey, 2017).

Oxidative stress, which results from the excess production of “reactive oxygen species (ROS),” has a fundamental role in the MTX-associated toxicities pathogenesis (Herman et al., 2005; Abbas et al., 2023b) and is related to its cytotoxic effects (Tunali-Akbay et al., 2010; Çağlar et al., 2013). Accumulated ROS upon MTX therapy attenuates antioxidant capacity in the liver and further attacks membrane lipids via lipid peroxidation, disrupting membrane integrity and eventually leading to tissue damage (Al Maruf et al., 2018; Jahovic et al., 2003; Ali et al., 2017).

The endoplasmic reticulum (ER) is a fundamental cellular organelle responsible for the newly synthesized proteins folding into their final 3-dimentional structures, post-translational modification, transportation, and quality control (Perri et al., 2016). In various pathological conditions implicating oxidative stress, ER functions are disturbed resulting in ER stress and initiation and propagation of unfolded protein response (UPR), which results in transient suppression of protein translation to allow recovery but leads to cell death when stress cannot be resolved (Soliman et al., 2019). Redox-controlled reversible modifications regulate UPR signaling, for example, thiol oxidation of ER molecular chaperones such as the binding immunoglobulin protein/78-kDa glucose-regulated protein (BiP/Grp78) that enhances the release of BiP from ER stress sensors and activates UPR (Wang et al., 2014). Another example is cysteine sulfhydration of protein tyrosine phosphatase 1B (PTP1B) that promotes phosphorylation and activation of double-stranded RNA-activated protein kinase-like ER kinase (PERK), a key ER stress sensors (Krishnan et al., 2011). In addition, ROS mediate cysteine sulfenylation of another ER stress sensor, inositol requiring enzyme 1α (IRE1α), which activates nuclear factor-E2-related factor-2 (Nrf2) to generate an antioxidative response (Hourihan et al., 2016). Further, one chaperone upregulated during the UPR is Protein Disulfide Isomerase (PDI), which is an essential redox-sensitive activator of PERK (Kranz et al., 2017). Despite that UPR helps cells to cope with ER stress, prolonged ER stress induces apoptotic cell death where the accumulation of unfolded protein in ER results in increased expression activating transcription factor 4 (ATF-4) that in turn increases the transcription of the proapoptotic protein, C/EPBα-homologous protein-10 (CHOP-10) (Li et al., 2014).

**FIGURE 1:**
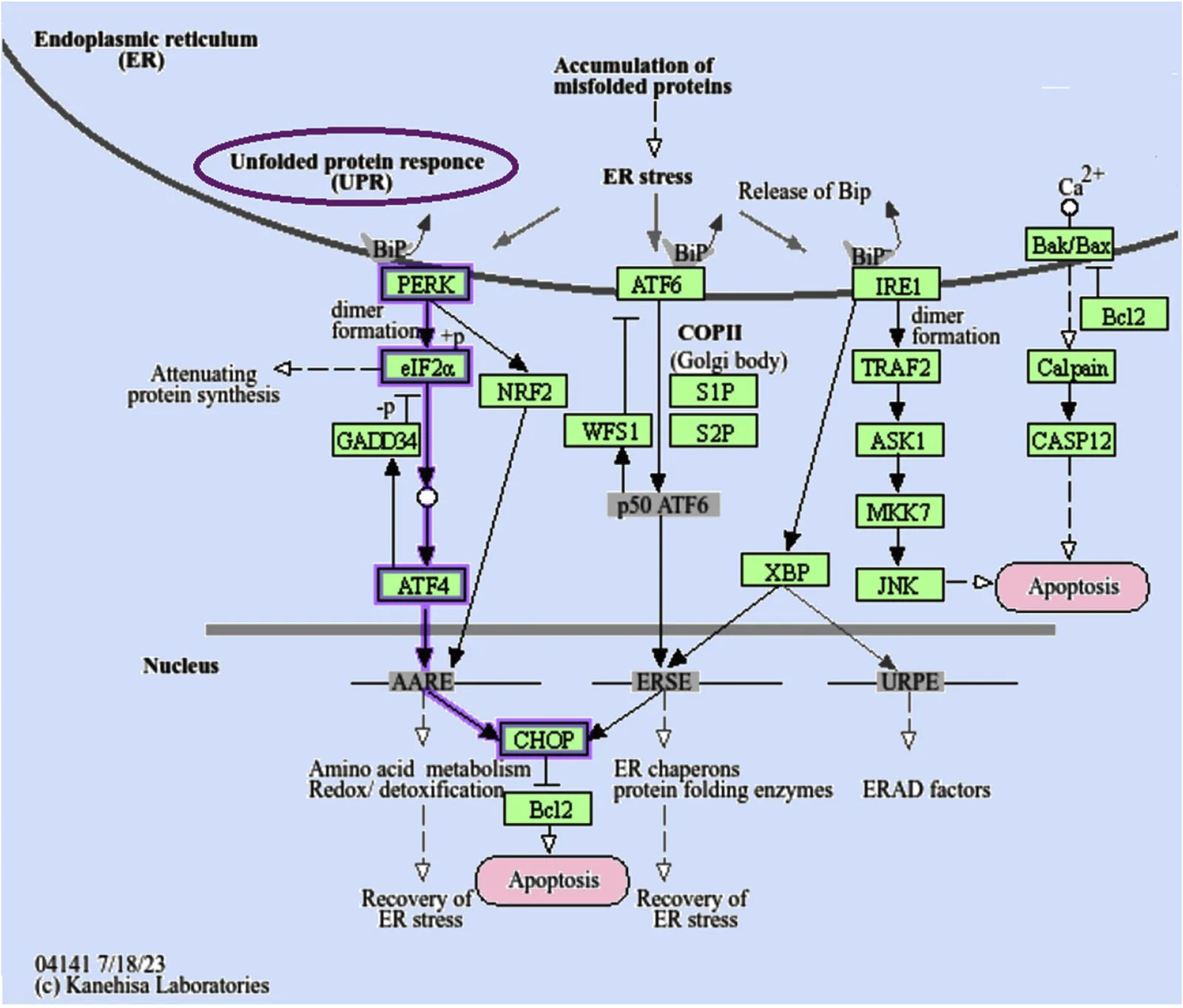
The endoplasmic reticulum (ER) serves as a cellular compartment where proteins undergo folding with lumenal chaperones. When misfolded proteins build up within the ER, this induces ER stress and triggers a signaling cascade known as the “unfolded protein response (UPR).” The “PERK/CHOP-10 pathway” is indicated by purple arrows. Nonetheless, in extreme conditions, the UPR’s protective measures are inadequate to reestablish proper ER function, leading to cellular apoptosis. “PERK, Protein Kinase R-like ER Kinase; eIF2α, eukaryotic translation initiation factor 2 subunit alpha; ATF4/6, activating transcription factor 4/6; S1/2P, membrane-bound transcription factor site-1/2 protease; CHOP, DNA damage-inducible transcript 3; IRE1, endoplasmic reticulum to nucleus signaling 1; TRAF2, TNF receptor-associated factor 2; ASK1, Apoptosis signal-regulating kinase 1; MKK7, mitogen-activated protein kinase kinase 7; XBP, X-box binding protein 1.” Adopted from (https://www.genome.jp/pathway/hsa04141+N01009) (last acceded 26 July 2024) (Kanehisa et al., 2016).

Apoptosis is essential in cellular homeostasis maintenance and is activated during adverse conditions (Elmore, 2007). Antitumor effects of MTX are, in part, attributed to apoptosis induction in many cancers (Pawlak et al., 2017; Wei et al., 2019). Unfortunately, MTX can trigger apoptosis in healthy tissues, including the liver (Abbas et al., 2023b). ROS signaling is crucial in mediating apoptosis induced by MTX, further deteriorating MTX-induced cytotoxicity (Xiong et al., 2020). Research has revealed that ER stress is associated with nephrotoxicity induced by MTX as well as hepatic stellate cells (HSCs) activation (Koo et al., 2016; Song et al., 2021). MTX-induced ER stress occurs predominantly through the PERK/CHOP-10 pathway (Koo et al., 2016) (Figure 1).

Nitazoxanide (NTZ) is commonly used to treat diarrhea caused by Giardia intestinalis (Abaza et al., 1998). It exhibits anti-infective, anti-inflammatory, and antineoplastic activities (Rossignol, 2014; Di Santo and Ehrisman, 2013; Shakya et al., 2018; Abd El-Fadeal et al., 2021). NTZ effectively reduces oxidative stress induced by the influenza-A virus (Huang et al., 2023) and mitigates proinflammatory cytokine release in macrophages triggered by lipopolysaccharide (Hong et al., 2012). Recently, NTZ has been repurposed to treat hyperlipidemia, hepatic steatosis (Li et al., 2022), as well as atherosclerosis (Ma et al., 2023). NTZ acts as a noncompetitive inhibitor of PDI; thus influencing PERK signaling (Piacentini et al., 2018).

The protective effects of NTZ against MTX-induced hepatotoxicity as well as the underlying mechanisms have not yet been explored previously. This study aims to investigate the hepatoprotective effects of NTZ utilizing a rat model of acute MTX-induced hepatotoxicity shedding some light on the mechanism of NTZ-mediated hepatoprotection with a focus on the possibility of modulation of oxidative stress, ER stress and apoptosis. The effects of NTZ were compared to those of the standard antioxidant, N-acetyl cysteine (NAC), as a well-documented hepatoprotective agent known to inhibit ER stress, and to those of 4-phenyl butyrate (4-PBA), a standard ER stress inhibitor (Soliman et al., 2019).

## 2 Materials and methods

### 2.1 The experimental animals

Currently, we included 36 adult male albino Wistar rats (with a body mass range equal to 190–230 g, 8–10 weeks of age) supplied by the “Faculty of Veterinary Medicine, Zagazig University.” Rats were kept in hygienic laboratory conditions, including temperature (22°C ± 3°C), relative humidity (60% ± 10%), and normal light/dark cycle. Rats were given unrestricted access to water and a standard diet *ad libitum*. The research protocol got approval from the Zagazig University Animal Care and Use Committee (code number ZU-IACUC/3/F/228/2022).

### 2.2 Drugs and chemicals

We purchased MTX (MYLAN) from Haupt Pharma GmbH, Germany), NTZ was purchased from Medizen Pharmaceutical Ind (Alexandria, Egypt), N-acetylcysteine (NAC) was obtained from SEDICO (Giza, Egypt), whereas Sigma–Aldrich company (MO, United States) provided Kolliphor^®^ EL and 4-Phenylbutyric acid (4-PBA). Microemulsions of 4-PBA were freshly prepared in a 1:1:9 solution of (Kolliphor^®^ EL (surfactant), ethanol, and saline). NTZ and NAC were dissolved in distilled H_2_O.

### 2.3 Experimental protocol and induction of hepatotoxicity

After an adaptation period of 2 weeks. We induced hepatotoxicity in rats by one MTX injection (20 mg/kg, i. p.) (Kalantar et al., 2019a) on day 7.

The animals were randomly divided into six experimental groups (six rats/group):

• CTRL group (rats were injected intraperitoneally with saline on day 7 plus Kolliphor^®^ EL from day 1- day 10),
• MTX group (rats were injected MTX intraperitoneally on day 7 plus Kolliphor^®^ EL throughout the experiment),
• NTZ-100 and NTZ-200 groups (rats were injected intraperitoneally with MTX on day 7 plus NTZ (100 and 200 mg/kg, daily) from day 1- day 10 by oral gavage, respectively, plus Kolliphor^®^ EL from day 1- day 10) (Elaidy et al., 2018; Li et al., 2022).
• NAC group (rats were injected intraperitoneally with MTX on day 7 plus NAC 500 mg/kg/day throughout the experiment by oral gavage, plus Kolliphor^®^ EL from day 1- day 10) (Li et al., 2018; Soliman et al., 2019).
• 4-PBA group (rats were injected intraperitoneally with MTX on day 7 plus 4-PBA (150 mg/kg) by intraperitoneal injection every other day (days 2, 4, 6, 8, and 10) (Soliman et al., 2019).

The administration of the drug began on the initial day of the experiment and was maintained for 10 days.

### 2.4 Blood and tissue collection

Sodium pentobarbital (50 mg/kg, i. p) was used for inducing anesthesia in rats (Mohamed et al., 2020), and blood samples were obtained from the retro-orbital plexus. The collected blood was centrifuged at 2,000 × g for 25 min at 4°C to separate serum samples, which were then stored at −80°C. Subsequently, the rats were euthanized by cervical dislocation. A laparotomy was performed to extract the liver, which was washed three times in saline solution and then dried. The liver was divided into portions: the first portion, from the largest hepatic lobule, was fixed in 10% formalin, while another portion, from a smaller lobule, was stored at −80°C. Later, liver homogenates were prepared in ice-cold phosphate-buffered saline and centrifuged at 2,000 × g for 25 min. The supernatants were then collected, aliquoted, and stored at −80°C.

### 2.5 Serum liver function parameters

For evaluating the liver function, serum levels of alanine transaminase (ALT)/aspartate transaminase (AST), and alkaline phosphatase (ALP) were measured using the Bio-Diagnostic colorimetric kits (Giza, Egypt).

### 2.6 Hepatic biomarkers assessment

#### 2.6.1 Oxidative stress biomarkers

Liver homogenates were utilized to assess hepatic malondialdehyde (MDA) and to evaluate the hepatic content of reduced glutathione (GSH) and superoxide dismutase (SOD) activity. Measurements were conducted using colorimetric kits (Bio-Diagnostic Co., Giza, Egypt).

#### 2.6.2 Proinflammatory cytokines

To evaluate hepatic inflammatory status in the liver homogenates, levels of TNF-α, IL-1β, and IL-6 were measured. The TNF-α levels were determined using the rat TNF-α ELISA kit from Boster Biological Technology (CA, United States), while IL-1β and IL-6 levels were measured using the rat IL-1 beta/IL-1F2 Quantikine ELISA Kit and the rat IL-6 Quantikine ELISA Kit from R&D Systems (MN, United States), respectively.

#### 2.6.3 Apoptotic biomarkers

Liver homogenates were used to quantify apoptosis markers. Bcl-2 and Bax levels were determined utilizing rat “B-cell CLL/lymphoma 2 and apoptosis regulator BAX ELISA” kits from CUSABIO (Wuhan, China). The Bax/Bcl-2 ratio was then calculated. Hepatic caspase-3 activity was measured utilizing a colorimetric assay kit purchased from Sigma Aldrich (MO, United States). Caspase-3 produces a colored end-product (p-nitroaniline, p-NA moiety). Detected P-NA is in direct proportion to caspase-3 concentration.

#### 2.6.4 ER-stress markers

“Inositol-requiring enzyme 1 (IRE1),” a marker of ER stress, was identified in the tissue homogenate utilizing a rat ELISA kit (Shanghai YL Biotech, Shanghai, China). We used the “quantitative reverse transcription-polymerase chain reaction (qRT-PCR)” to measure the relative mRNA expressions of activating transcription factor-4 (ATF4), CHOP-10, Glucose-Regulated Protein 78 (GRP78), and PERK as markers for ER stress. Frozen hepatic samples were lysed using Qiazol reagent, and total RNA extraction was done with a QIAGEN RNAeasy Mini Kit (Hilden, Germany). For each sample, 10 ng of total RNA was used to synthesize cDNA with Quantiscript reverse transcriptase from the QuantiTect Reverse Transcription Kit (QIAGEN, Hilden, Germany). The cDNA was amplified using a Syber Green I PCR-Master Kit from Fermentas (PA, United States). Table 1 provides the sequences of the gene primers. The cycle threshold (CT) values of gene expression were normalized to the housekeeping gene β-actin, and the expression ratios were calculated using the ΔΔCT method.

**TABLE 1:** The sequence of the primers used in the qRT-PCR experiment.

Target gene	Primer sequence: 5′–3′	Accession number	References
ATF4	F: TCCTCGATACCAGCAAATCC R: ACCCATGAGGTTTGAAGTGC	NM_024403.2	Soliman et al. (2019)
CHOP10	F: AGGTCCTGTCCTCAGATGAAA R: TAGGGATGCAGGGTCAAGAGT	NM_024134.2	Soliman et al. (2019)
GRP78	F:5′-GAC GCA CTT GGA ATG ACC CTT-3 R: 5′-TTGGTT TGC CCA CCT CCG AT-3′	NM_013083.2	Liu et al. (2018)
PERK	F: 5′- GCCACAGAGAACAGCGTGTA-3′ R: 5′-GATCGTCGGCAGAGGAATAA-3′	NM_031599.2	Chen et al. (2023)
β-actin	F: ATGGATGACGATATCGCTGC R: CTTCTGACCCATACCCACCA	NM_031144.3	Soliman et al. (2019)

#### 2.6.5 Determination of the protein levels of endoplasmic reticulum stress markers by Western blotting and ELISA

Protein levels of phosphorylated PERK and CHOP-10 proteins were determined in the homogenate of the liver tissue. Proteins were denatured in Laemmli buffer. Subsequently, the protein samples in equal volume were run on a 13%–15% sodium dodecyl sulfate (SDS) polyacrylamide gel electrophoresis, then moved to nitrocellulose membranes. The membrane was blocked in tris-buffered saline with Tween 20 (TBST) and 3% bovine serum albumin (BSA) at room temperature for 1 h. The blots were probed with the primary antibodies specific for phosphorylated PERK and CHOP (Thermo Fischer Scientific Inc.) at 4°C overnight, then the blots were rinsed 3–5 times for 5 min with TBST and incubated with the horseradish peroxidase (HRP)-conjugated anti-rabbit IgG Secondary Antibody (Thermo Fischer Scientific Inc.) against the blotted target protein for 1 h at room temperature. The chemiluminescent substrate (ThermoFischer Scientific Inc.) was applied to the blot according to the manufacturer’s recommendation. The chemiluminescent signals were captured using a CCD camera-based imager. Image analysis software was used to read the band intensity of the target proteins against the control sample after normalization by β-actin (El-Sherbeeny et al., 2020) on the Chemi Doc MP imager. In addition, ELISA kits were used to determine the level of ATF4 and GRP78.

### 2.7 Histopathological analysis

Hepatic injury can be detected by hematoxylin and eosin (H&E) staining of liver tissues (Mousa et al., 2022; Bilasy et al., 2015).

The formalin-fixed liver specimens were inserted in liquified paraffin wax and cut into 5-μm thick sections utilizing a Leica microtome (Leica RM 2155, England). Xylene was applied to paraffinized sections to dewax them. The treated sections were then gradually hydrated and subjected to hematoxylin and eosin (H&E) staining. The sections were blindly screened, examined for histopathological alterations, and scored by an experienced pathologist. Images were captured by a LEICA light microscopy (LEICA ICC50 W, England). Scoring was done according to congestion, inflammatory cell infiltration, and liver cell necrosis. Each variable was scored for each sample slide, and we used a common scoring system where [0] = no alteration, [1] = mild changes, [2] = moderate changes, and [3] = severe changes (Kalantar et al., 2019b; Moodi et al., 2020).

### 2.8 Immunohistochemical analysis

Paraffin sections from different groups of liver tissues from rats were stained by immunohistochemistry (IHC) according to the manufacturing protocol using Anti-caspase 3 antibody (ab4051), at dilution 1:1,000, Anti-Bax antibody (ab32503), at dilution 1:250 and Anti-Bcl-2 antibody (ab182858), at dilution 1:500, (abcam, Cambridge, United Kingdom). The tissue sections from all experimental groups were dewaxed and hydrated (Alomar et al., 2021). Staining was then performed using the DAB chromogenic agent (Expose mouse and rabbit specific HRP/DAB detection kit, Abcam; Ready-to-use; Cat. #: ab80436). Counterstaining by hematoxylin was done as a final step (Elsherbiny et al., 2019). All photos of the tissue sections stained by IHC were captured using a Swift microscope associated with a Swift digital camera. For quantitative analysis, we selected five representative areas from each group. If a tissue section had areas with both low abundance and high abundance of stained cells, both areas were selected as representative areas and included in the analysis. Individual cells were identified by strong brown stain and manually counted. The cell counting was repeated three times for each area. All images were analyzed in a blinded fashion.

### 2.9 Data analysis

We used the GraphPad Prism, version 9.1.0 (221) (CA, United States) in statistical analysis. Data of the study were displayed as mean ± standard error of the mean (SEM). For determining the inter-group variations, we applied the one-way ANOVA test followed by Tukey’s *post hoc* test for multiple comparisons. Kruskal–Wallis and Dunn’s tests were used for analysis and pair-wise comparison of histopathology scoring. A significant difference was assumed if *p*-values were <0.05.

## 3 Results

### 3.1 Nitazoxanide improved liver function in rats with MTX-hepatotoxicity

**FIGURE 2:**
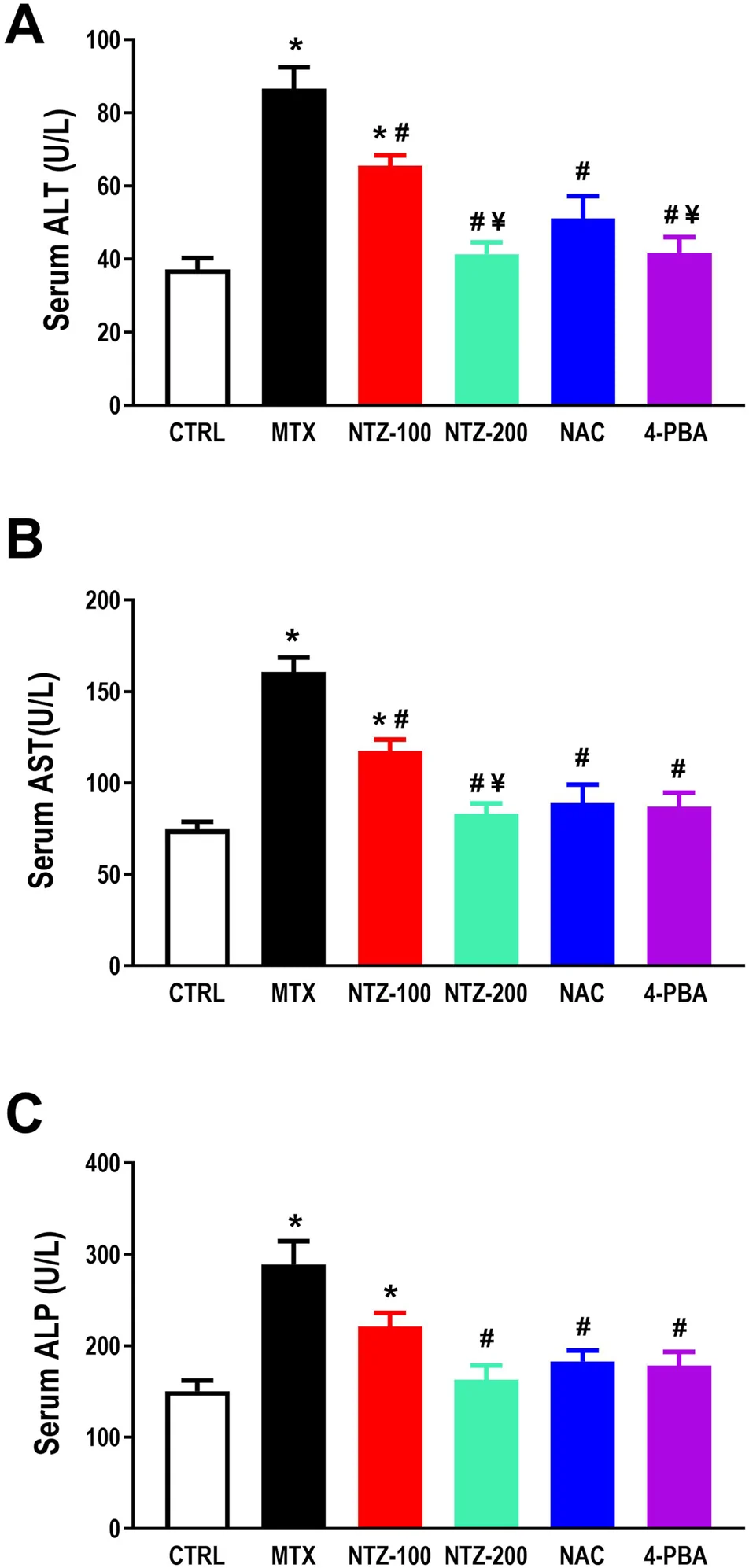
Effect of 10 days administration of nitazoxanide (NTZ-100 and NTZ-200), N-acetylcysteine (NAC, 500 mg/kg), and 4-Phenylbutyric acid (4-PBA) on liver function tests. Liver function is expressed as serum levels of **(A)** ALT, **(B)** AST, and **(C)** ALP. The data presented here are mean ± SEM. * Vs. CTRL; # Vs. MTX group; ¥ Vs. NTZ-100 group, *p* < 0.05.

As depicted in Figure 2, an impaired liver function was observed in the MTX group compared to CTRL, as indicated by significantly elevated serum liver enzymes. NTZ reduced the elevation in both ALT and AST in a dose-dependent way (Figures 2A, B), whereas a significant reduction in ALP (Figure 2C) was observed with the higher dose (NTZ-200) when compared with the MTX group. Noteworthy, NTZ-200-induced reductions of liver enzymes reached closer levels to those observed with either NAC or 4-PBA.

### 3.2 Nitazoxanide lessened oxidative stress in rats with MTX-hepatotoxicity

**FIGURE 3:**
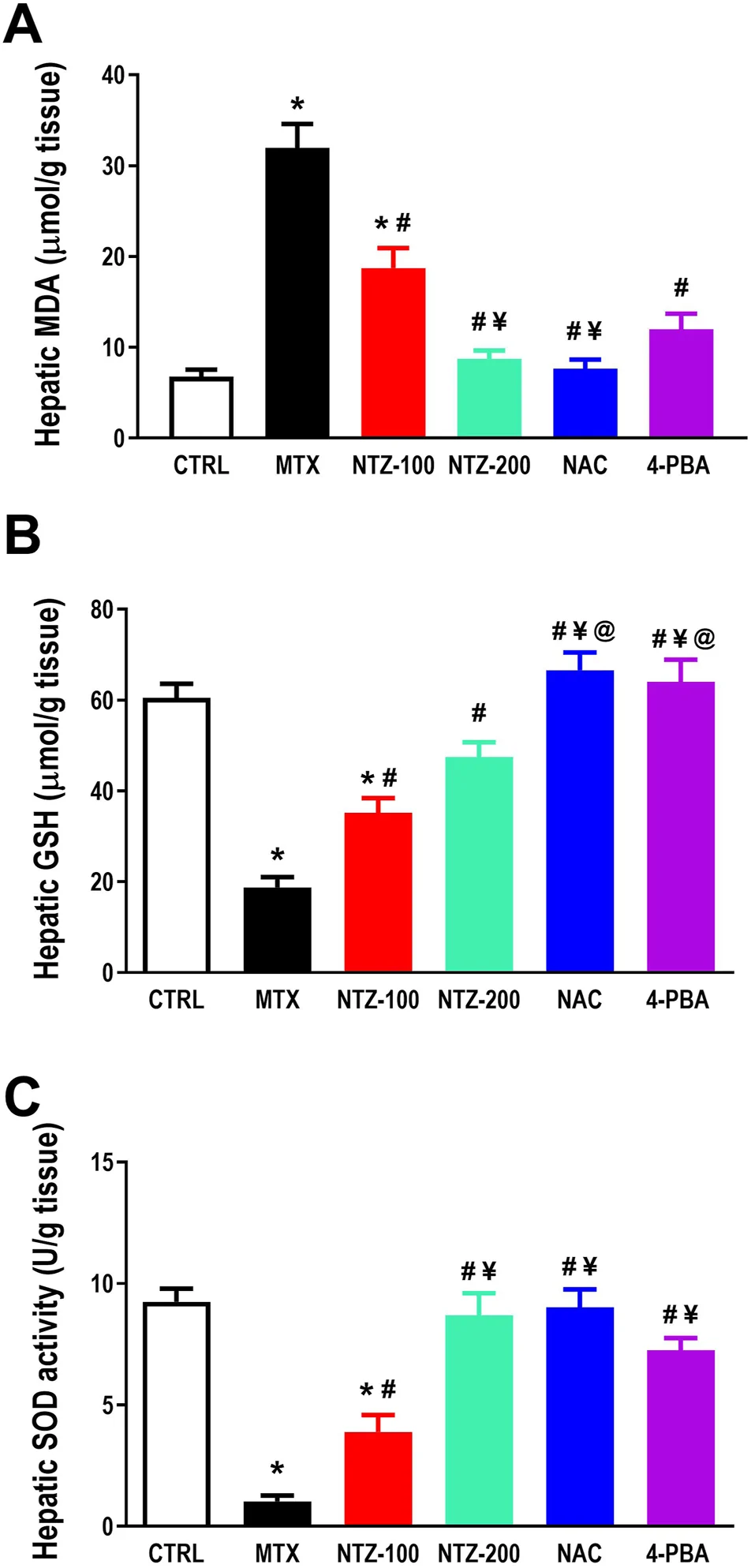
Effect of 10 days administration of nitazoxanide (NTZ-100 and NTZ-200), N-acetylcysteine (NAC, 500 mg/kg), and 4-Phenylbutyric acid (4-PBA) on hepatic oxidative stress. Hepatic oxidative stress is demonstrated through **(A)** increased hepatic MDA, **(B)** reduced hepatic reduced glutathione (GSH), and **(C)** superoxide dismutase activity (SOD, **(C)**. Data are means ± SEM. ** Vs. CTRL; # Vs. MTX group; ¥ Vs. NTZ-100 group, *p* < 0.05.

As an indicator for lipid peroxidation, hepatic MDA was determined, and a significant rise in hepatic MDA was found in the MTX group as compared to CTRL. NTZ dose-dependently alleviated MTX-induced increase in hepatic MDA, indicating antioxidant properties. NTZ-200 exhibited a better effect than NTZ-100 and resembles the effect of both NAC and 4-PBA (Figure 3A). Further, on evaluating hepatic antioxidant capacity, it was significantly attenuated in MTX group in comparison with CTRL as demonstrated by the significantly lower hepatic GSH and SOD activity (Figures 3B, C). NTZ at both doses significantly enhanced hepatic GSH levels as well as SOD activity with a more pronounced effect with the higher dose, compared to the MTX group. NTZ-200 was close to NAC and 4-PBA effects concerning hepatic SOD activity (Figure 3C), yet it was less effective than both concerning hepatic GSH (Figure 3B).

### 3.3 Nitazoxanide reduced inflammation in rats with MTX-hepatotoxicity

**FIGURE 4:**
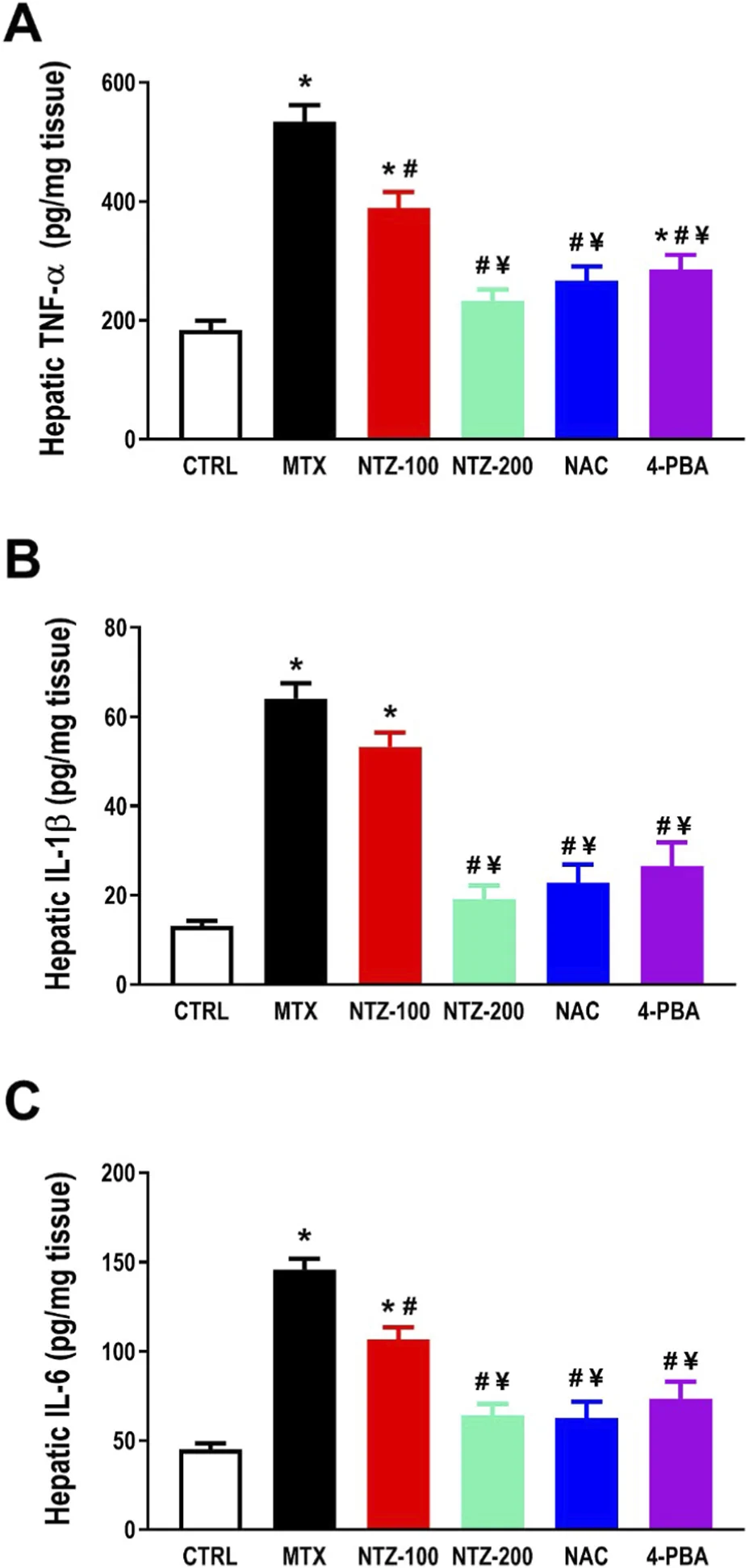
Effect of 10 days administration of nitazoxanide (NTZ-100 and NTZ-200), N-acetylcysteine (NAC, at 500 mg/kg), and 4-Phenylbutyric acid (4-PBA) on hepatic inflammation. Hepatic inflammation is demonstrated by increased hepatic levels of TNF-α, **(A)**, IL-1β, **(B)** and IL-6, **(C)**. Data presented here are mean ± SEM. * Vs. CTRL; # Vs. MTX group; ¥ Vs. NTZ-100 group, *p* < 0.05.

A profound hepatic inflammation was observed in the MTX group, as demonstrated by significant augmentation in hepatic TNF-α, IL-1β, and IL-6 versus the CTRL group (Figures 4A–C). NTZ significantly ameliorated hepatic inflammation by reducing the levels of the proinflammatory cytokines compared to the MTX group in a dose-dependent manner. NTZ-200 approaches the levels reached by both NAC and 4-PBA (Figures 4A–C).

### 3.4 Nitazoxanide reduced apoptosis in rats with MTX-hepatotoxicity

**FIGURE 5:**
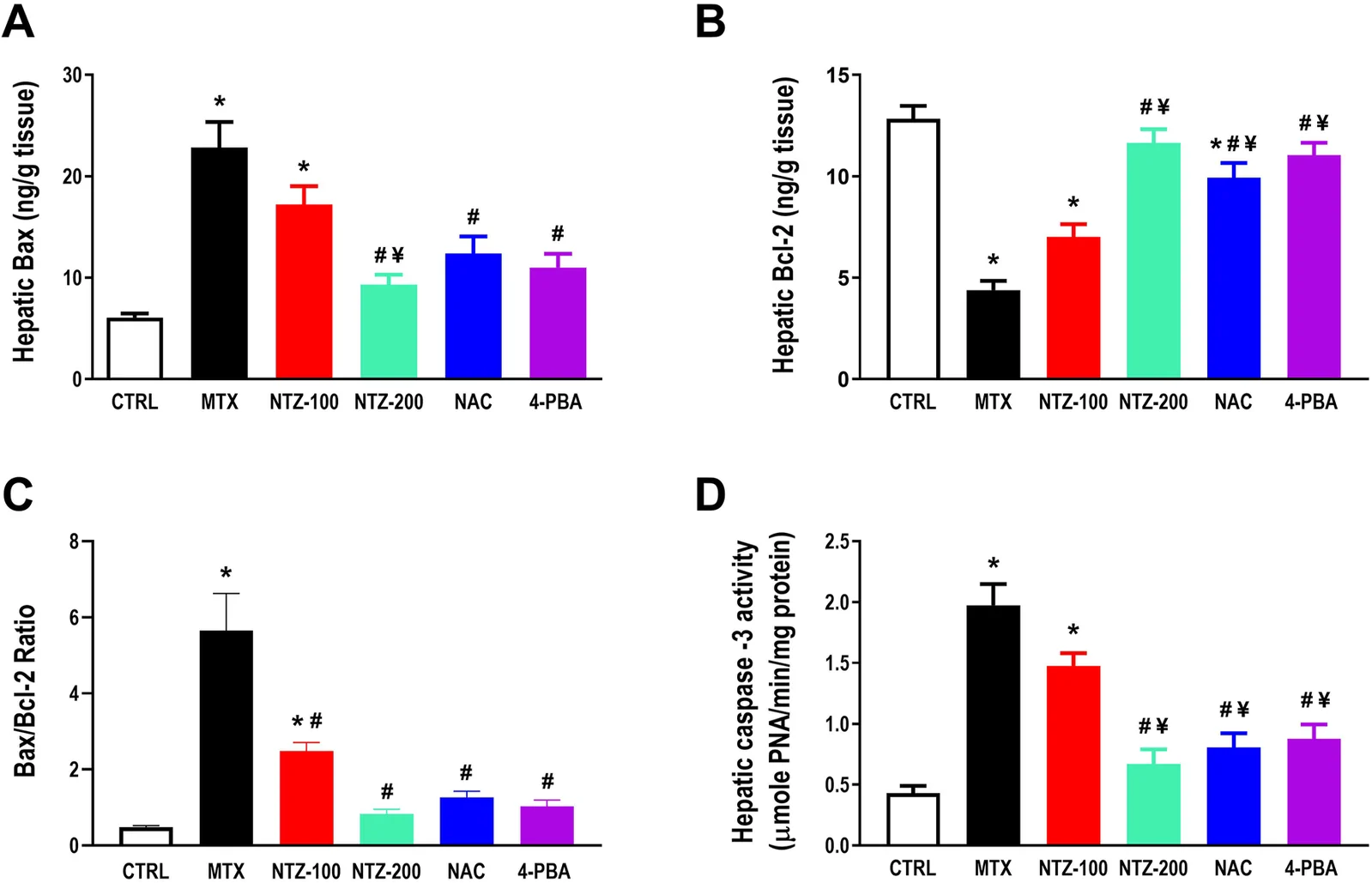
Effect of 10 days administration of nitazoxanide (NTZ-100 and NTZ-200) at 100 and 200 mg/kg/day, N-acetylcysteine (NAC, 500 mg/kg), and 4-Phenylbutyric acid (4-PBA) on hepatic apoptosis. Hepatic apoptosis is expressed by hepatic levels of Bax **(A)**, Bcl-2 **(B)**, Bax/Bcl-2 ratio **(C)**, and caspase-3 activity **(D)**. Data presented here are means ± SEM. * Vs. CTRL; # Vs. MTX group; ¥ Vs. NTZ-100 group, *p* < 0.05.

MTX significantly induced hepatic apoptosis as demonstrated by increased proapoptotic markers, Bax level, and caspase-3 activity (Figures 5A, D) while reduced antiapoptotic Bcl-2 level (Figure 5B) and increased Bax/Bcl-2 ratio (Figure 5C) compared to CTRL group. MTX-induced apoptosis was significantly attenuated with all treatments except for NTZ-100, where, as depicted in Figures 5A–D, Bcl-2 level was augmented while Bax was reduced, and consequently, Bax/Bcl-2 ratio was also reduced as well as caspase-3 activity all when compared to MTX group. NTZ effects were dose-dependent, where the higher dose was more efficient and closer to the effect of NAC or 4-PBA.

### 3.5 Nitazoxanide reduced the mRNA expression of ER stress markers in rats with MTX-hepatotoxicity

**FIGURE 6:**
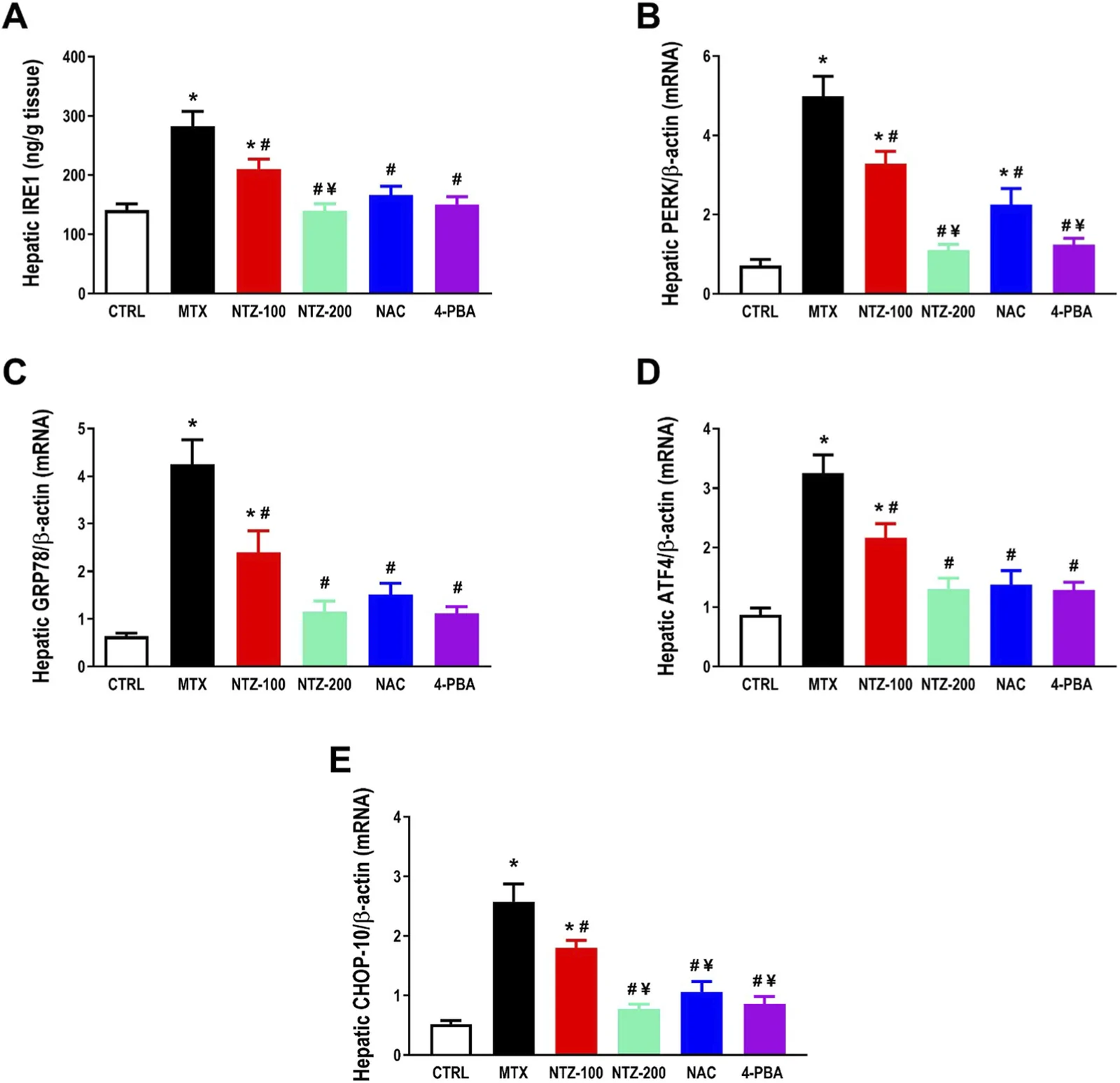
Effect of 10 days administration of nitazoxanide (NTZ-100 and NTZ-200) at 100 and 200 mg/kg, respectively, N-acetylcysteine (NAC) at 500 mg/kg and 4-Phenylbutyric acid (4-PBA) on hepatic endoplasmic reticulum (ER) stress. Hepatic ER-stress is expressed by hepatic levels of stress sensor protein, inositol-requiring enzyme 1 IRE1, **(A)**, and hepatic mRNA expression of PERK, **(B)**, GRP78, **(C)**, ATF4, **(D)**, and C/EBP homologous protein CHOP-10, **(E)**. Data are means ± SEM. * Vs. CTRL; # Vs. MTX group; ¥ Vs. NTZ-100 group, *p* < 0.05.

To determine whether the hepatoprotective effects of NTZ against MTX-hepatotoxicity are associated with ER stress reduction, mRNA expression and the protein level of ER stress markers were determined in liver homogenates. As depicted in Figure 6A, the MTX group exhibited a significant increase in hepatic IRE1 content, a stress sensor protein, compared to CTRL. Further, MTX increased the expression of PERK (Figure 6B), GRP78 (Figure 6C), ATF4 (Figure 6D), and CHOP-10 (Figure 6E) in the liver compared to CTRL, indicating that ER stress is implicated in MTX-induced hepatotoxicity. Hepato-protectant (NAC) and ER stress inhibitor (4-PBA) significantly downregulated ER stress markers in the liver compared to the MTX group, as depicted in Figures 6A–E. NTZ significantly attenuated hepatic ER stress induced by MTX, as demonstrated by the downregulation of ER stress markers compared to the MTX group (Figures 6A–E). NTZ effects were dose-related, and the higher dose was efficient enough to replicate the 4-PBA effect on ER stress markers.

### 3.6 Nitazoxanide reduced protein levels of ER stress markers in rats with MTX-hepatotoxicity

**FIGURE 7:**
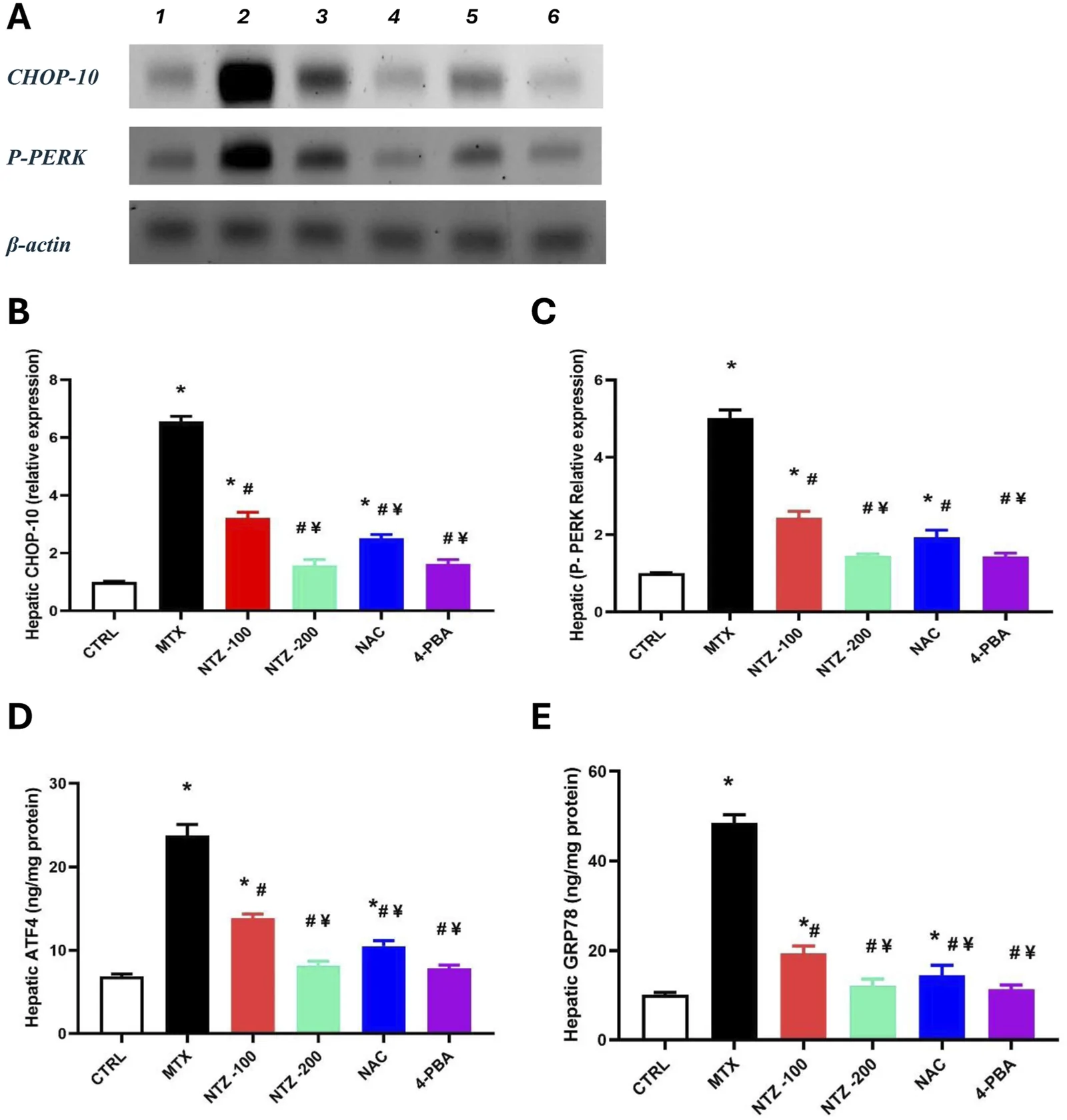
Effect of nitazoxanide on hepatic markers of endoplasmic reticulum stress. **(A)** Cropped Western blot gels for CHOP-10 and P-ERK proteins in the experimental groups (1) CNTR, (2) MTX, (3) MTX + NTZ-100, (4) NTZ-200 groups, MTX + NAC and MTX + 4-PBA. **(B, C)** Column charts for CHOP-10 and P-PERK levels. **(D, E)** Column charts for ATF4 and GRP78 levels. Data are means ± SEM. * Vs. CTRL; Vs. MTX group; ¥ Vs. NTZ-100 group, *p* < 0.05.

Figure 7A demonstrates the cropped WB gels for experimental groups. Panels 7B and 7C show the protein levels for CHOP-10 and P-PERK, which were significantly increased in the MTX group versus the CTRL group. Rats who received NTZ-100 or NTZ-200 mg/kg showed dose-dependent declines in these 2 markers. In addition, the levels of ATF4 and GRP78 are shown in (Figures 7D, E). The MTX group showed significant elevations in hepatic ATF4 and GRP78 versus the CTRL group. NTZ-100 or NTZ-200 mg/kg groups produced dose-dependent suppressions in these 2 markers (Figures 7D, E). Further, NAC and 4-PBA groups suppressed the ER stress markers in a significant manner (Figures 7B–D).

### 3.7 Histopathology findings

**FIGURE 8:**
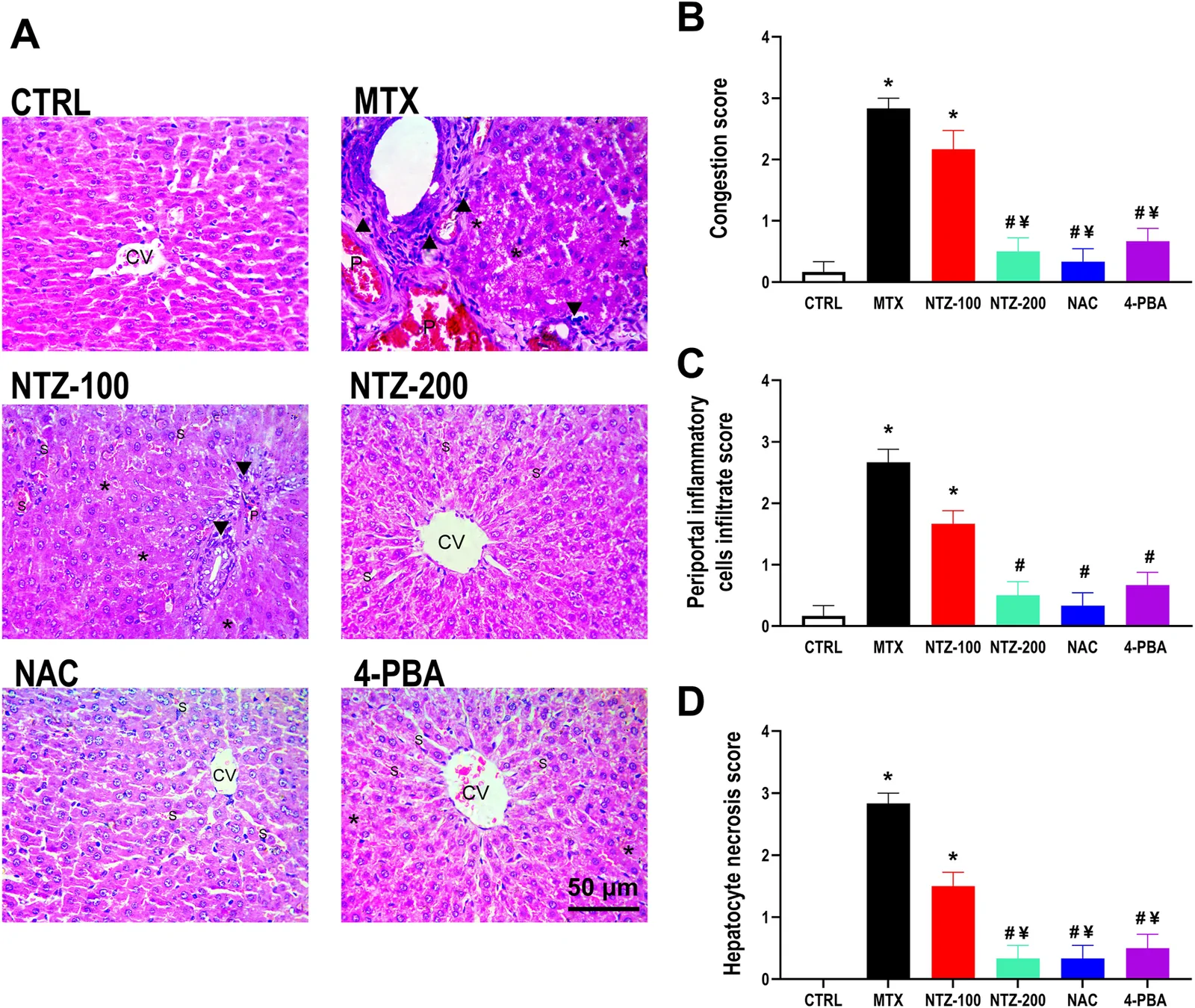
Effect of 10 days administration of nitazoxanide (NTZ-100 and NTZ-200), N-acetylcysteine (NAC) at 500 mg/kg, and 4-Phenylbutyric acid (4-PBA) on hepatic histopathological alterations. **(A)** Photomicrograph (H&E ×400) of rat livers from different study groups, CTRL group showing a normal architecture of liver tissue with normal central vein (CV). MTX group showing marked portal congestion (P), marked periportal inflammatory infiltrate (arrowheads), and moderate hepatocyte necrosis (asterisks). NTZ-100 group showing moderate hepatoportal (P) and sinusoidal congestion (S), moderate periportal inflammatory infiltrate (arrowheads), and moderate hepatocyte necrosis (asterisks). NTZ-200 group showing the normal architecture of liver tissue with mild sinusoidal congestion (S) Central vein (CV). NAC group showing preserved hepatic architecture with mild sinusoidal congestion (S). Central vein (CV). 4-PBA group showing preserved architecture with mild sinusoidal congestion (S). Central vein (CV). The right panels represent histopathological scoring expressed as congestion score **(B)**, periportal inflammatory cells infiltrate score **(C)**, and hepatocyte necrosis score **(D)**. Data are means ± SEM. * Vs. CTRL; # Vs. MTX group; ¥ Vs. NTZ-100 group, *p* < 0.05.

Figure 8A shows the features of MTX-induced hepatopathy detected in the liver sections of MTX rats. Livers appeared in a disarrayed pattern with numerous structural abnormalities that can be observed compared to the CTRL rats. Such alterations were depicted as remarkable portal congestion, periportal inflammatory infiltrate, and moderate hepatocyte necrosis (nuclear pyknosis, karyorrhexis, and karyolysis with cytoplasmic eosinophilia), whereas the CTRL group exhibited normal architecture of liver tissue. Histopathological scoring revealed significant portal congestion, periportal inflammatory infiltrate, and hepatocyte necrosis compared to CTRL (Figures 8B–D). NTZ at 100 mg/kg failed to alleviate hepatic histopathology scoring significantly (Figures 8B–D), which is in alignment with the features displayed in photomicrographs of H&E-stained liver sections where moderate hepatoportal and sinusoidal congestion, moderate periportal inflammatory infiltrate, and moderate hepatocytes necrosis were found. On the other hand, the higher dose of NTZ (200 mg/kg) as well as NAC both depicted preserved architecture with mild sinusoidal congestion (Figure 8A) and significantly alleviated histopathological scoring (Figures 8B–D). The 4-PBA group showed mild sinusoidal congestion with focal hepatocyte necrosis as manifested by nuclear pyknosis, karyorrhexis, and karyolysis with cytoplasmic eosinophilia (Figure 8A), yet it significantly attenuated histopathological scoring compared to MTX-group (Figures 8B–D).

### 3.8 Immunohistochemical findings

**FIGURE 9:**
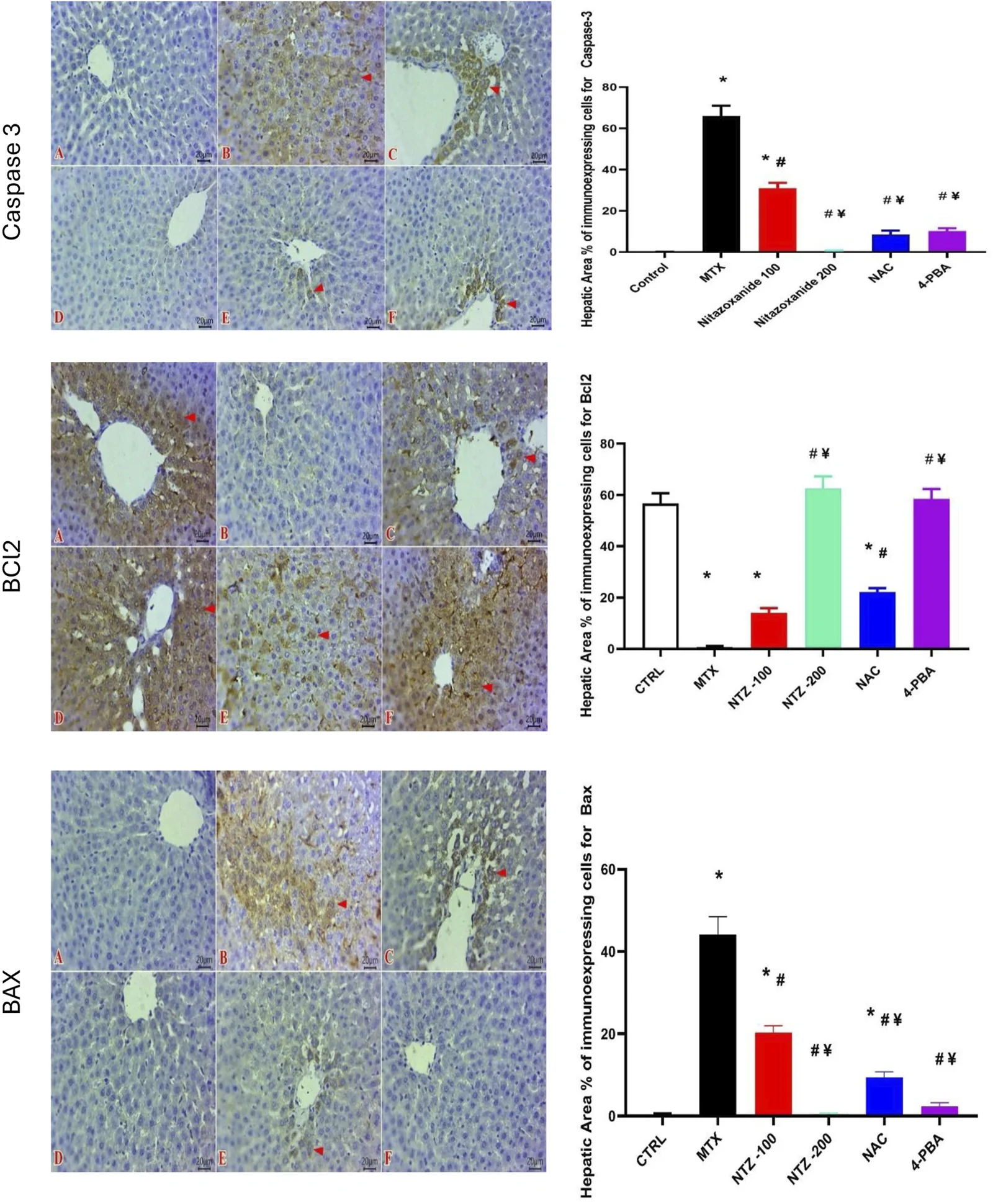
Effect of 10 days administration of nitazoxanide (NTZ-100 and NTZ-200), N-acetylcysteine (NAC) at 500 mg/kg, and 4-phenylbutyric acid (4-PBA) on hepatic immunohistochemical alterations. The left panels are for **Caspase 3**: Representative photomicrographs of immunostained liver sections for Caspase 3 (Scale bar 20 μm) showing: non-detectable expressed cells in CTRL group (A), and NTZ-200 group (D). Numerous positive expressed hepatic cells in MTX group (B). moderate number of stained cells in NTZ-100 group (C). Few labeled cells at both the NAC group (E) and 4-PBA group (F). IHC counterstaining with Mayer’s hematoxylin. (Arrowheads refer to positive expressed cells that reveal a golden brown color), **Bcl-2:** Representative photomicrographs of immunostained liver sections for Bcl-2 (Scale bar 20 μm) showing marked cytoplasmic expressions within abundant hepatocytes in CTRL group (A), NTZ-200 group (D) and 4-PBA group (F). Negative expressions of Bcl-2 in MTX group (B). Few numbers of expressed hepatocytes in the NTZ-100 group (C). Moderate number of expressed cells in NAC group (E). IHC counterstaining with Mayer’s hematoxylin (Arrowheads refer to positive expressed cells that reveal a golden brown color) and **BAX:** Representative photomicrographs of immunostained liver sections for Bax (Scale bar 20 μm) showing: no expression in CTRL group (A), NTZ-200 group (D), and 4-PBA group (F). intense numbers of positive labeled hepatocytes in MTX group(B). Moderate number of positive cells at NTZ-100 group (C). Few immuno-labeled cells containing Bax expression in the NAC group (E). IHC counterstaining with Mayer’s hematoxylin. (Arrowheads refer to positive expressed cells that reveal a golden brown color). **The right panels** represent the hepatic area % of immunoexpressing cells for caspase-3, BCl2, and BAX. Data are means ± SEM. * Vs. CTRL; # Vs. MTX group; ¥ Vs. NTZ-100 group, *p* < 0.05.

The immunohistochemical investigation demonstrated a significant increase in expression of proapoptotic makers, Bax, and caspase-3, in MTX treated group, which was associated with decreased antiapoptotic marker, Bcl-2, expression when compared to the CTRL group (Figure 9). Immunohistochemical analysis also revealed a significant decrease in the hepatic expression of both proapoptotic markers Bax and caspase-3 with significant increase in antiapoptotic Bcl-2 expression in rats treated with NTZ-200, NAC or 4-PBA when compared to MTX group (Figure 9).

## 4 Discussion

**FIGURE 10:**
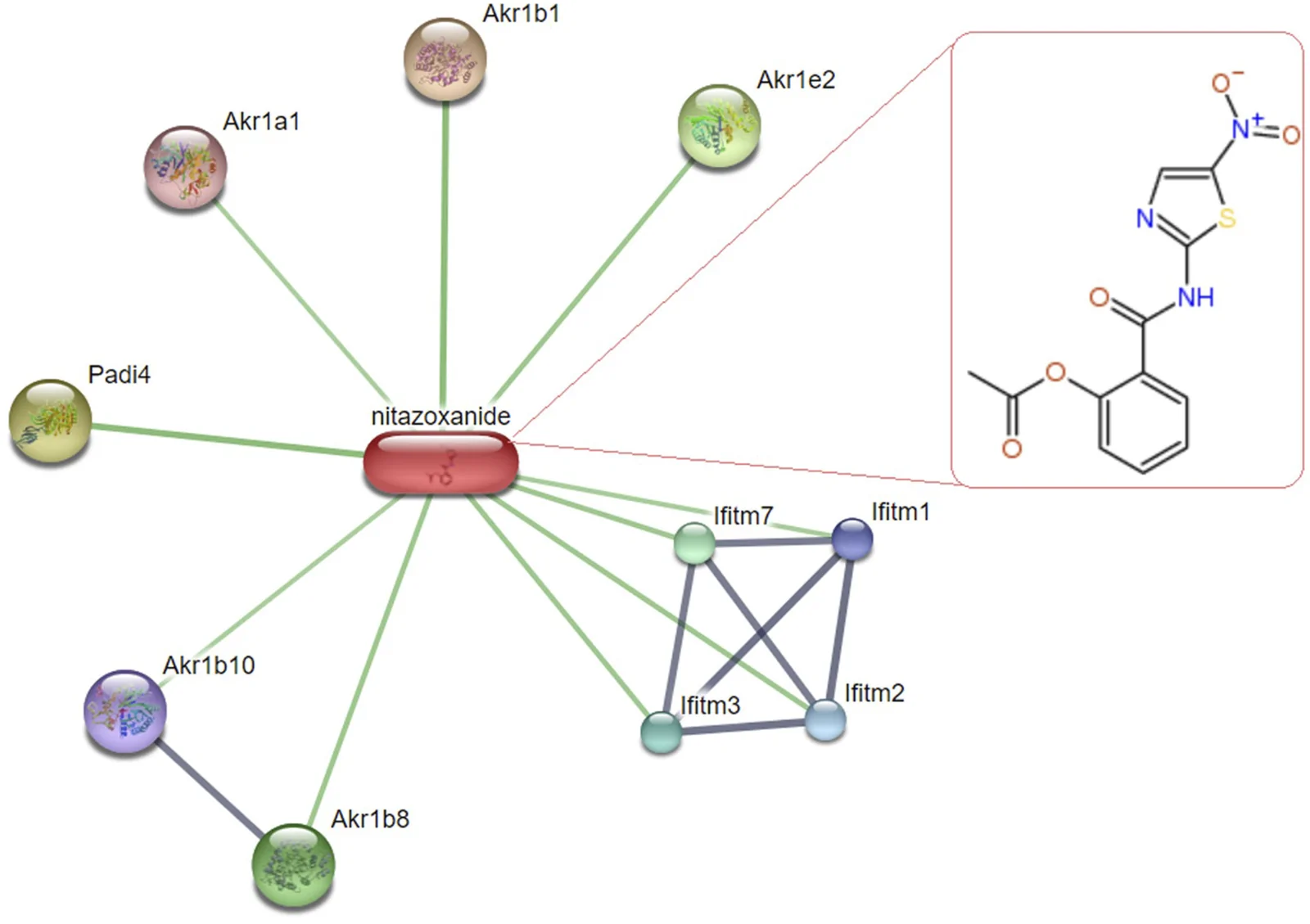
Network of predicted protein-interaction of Nitazoxanide (NTZ) in “*Rattus norvegicus*,” a visual depiction of the potential protein associates of NTZ identified through the “STITCH database (http://stitch.embl.de/) (last accessed 27 July 2024).” The thickness of the interconnected lines indicates the strength of evidence for each interaction, “protein-protein interactions are depicted in grey, while chemical-protein interactions are indicated in green.” This network provides additional support for the proposed functions of NTZ in influencing cellular processes associated with oxidative stress, inflammation, and apoptosis. The upper right chemical structure (nitrothiazole and salicylamide) pertains to the NTZ. Abbreviations: “Akr1b1, Aldose reductase; Padi4, Protein-arginine deiminase type-4; Akr1e2, 1,5-anhydro-D-fructose reductase; Akr1b8, aldose reductase-related protein 2; Ifitm7, Protein Ifitm7; Ifitm 1/2/3, Interferon-induced transmembrane protein 1/2/3, the Ifitm 3 may inhibit early replication of influenza A virus; Akr1b10, Aldo-keto reductase family 1, member B10; Akr1a1, Alcohol dehydrogenase [NADP (+)].”

Liver toxicity is a severe outcome of MTX therapy, limiting its clinical use and contributing to its related morbidity and mortality (Conway and Carey, 2017; Abbas et al., 2023b). Therefore, searching for potential adjuvant therapies with hepatoprotective capacities to be used during chemotherapy is widely adopted (Liang et al., 2021) with the need to understand the mechanisms underlying MTX-induced liver toxicity. In this study, we demonstrated the hepatoprotective effect of NTZ, an antiprotozoal drug, against MTX-induced hepatotoxicity. Challenging rats with MTX evoked hepatotoxicity as manifested by elevated liver function enzymes in serum and disarrayed hepatic histopathologic features, which aligns with previous reports (Karabulut et al., 2020; Morsy et al., 2022). Pretreatment with NTZ protects the liver against MTX-induced injury as depicted by lower liver enzyme levels in serum and nearly normal histological features, especially with the higher dose; this is following previous reports on NTZ-ameliorative effects on liver injury in CCl4-induced hepatopathy and in steatohepatitis models (Walczak et al., 2018; Li et al., 2022; Odagiri et al., 2021; Vanessa Legry et al., 2022). Substantial evidence indicates that MTX-induced toxicities implicate oxidative stress (Cetin et al., 2008; Abbas et al., 2023b; Abbas et al., 2023a) and exhaustion of antioxidant defenses, like downregulation of GSH and the “Nrf2/heme oxygenase-1” signaling (Bu et al., 2018). This supports our findings where MTX injection evoked oxidative stress with augmented hepatic lipid peroxidation product while declining hepatic antioxidant capacity as depicted by lower GSH and SOD. NTZ significantly alleviated MTX-induced oxidative stress, which can be attributed to the antioxidant properties of its structural moieties (nitrothiazole and salicylamide) (Hemphill et al., 2006; Elazar et al., 2009). Our findings are consistent with the oxidative stress suppressive effect of NTZ that underpins its antiviral effect against the influenza-A virus (Huang et al., 2023) and the chemoprophylactic action in breast cancer (Pal et al., 2020). Furthermore, using the “STITCH database (http://stitch.embl.de/) (Kuhn et al., 2007),” authors evaluated known/predicted protein interactions with NTZ. Our findings revealed a network of interactions in which NTZ may be connected to various proteins critical for oxidative stress, inflammation, and apoptosis (Khayami et al., 2020; Zhou et al., 2018; Guo et al., 2024; Rajak et al., 2022) (Figure 10).

Oxidative stress, associated with high dose MTX, triggers a systemic inflammatory response where proinflammatory cytokines further contribute to tissue damage (Kalantar et al., 2019a; Hussain et al., 2016); this reinforces the results of the current study where elevated TNF-α, IL-1β, and IL-6 were noticed in the liver following MTX challenge. Our findings revealed an inflammatory repressing effect of NTZ. Several studies have proven the anti-inflammatory potential of NTZ as it downregulates proinflammatory cytokines in LPS-induced systemic inflammation (Vanessa Legry et al., 2022), ovariectomized mice (Li et al., 2021), and type-2 diabetic patients (Castillo-Salazar et al., 2021) which supports our findings. Generated ROS during MTX administration provoke DNA damage and induce apoptosis via activating intrinsic apoptotic pathways, as documented in various studies (Koppelmann et al., 2012; Morsy et al., 2022). In our experiment, the MTX group showed upregulated caspase 3 and the proapoptotic protein, Bax, while downregulated Bcl-2. Hence, we can conclude that these actions may contribute to MTX-induced hepatotoxicity.

During stressful conditions, such as MTX-induced oxidative damage, unresolved ER stress leads to the accumulation of unfolded proteins promoting apoptosis (Teske et al., 2011). Activated UPR can restore ER homeostasis via integrated intracellular signaling (Hetz, 2012). ER stress triggers three branch pathways of the UPR, involving activation of transcription factor 6 (ATF6) (Shen et al., 2005), IRE1 (Chen and Brandizzi, 2013), and PERK (Figure 1). During ER stress, GRP78, the primary regulator of UPR, dissociates from the ER compartment, activating the main PERK protein pathway. On the other hand, PERK induces translation initiation factor eIF2α phosphorylation (Han et al., 2013), which activates (ATF4) and CHOP and eventually culminates in apoptotic cell death. The “PERK/CHOP-10” pathway serves as a significant mediator for cell survival (Han et al., 2013). The response to ER stress plays a pivotal role in MTX-induced apoptosis, where MTX primarily triggers ER stress via the “PERK/CHOP” pathway (Song et al., 2021). In the present investigation, MTX challenge induced ER stress and UPR mainly through the “PERK/CHOP” pathway, consistent with previous studies on MTX-induced hepatic injury (Schmidt et al., 2022), nephrotoxicity (Song et al., 2021), and cognitive impairment (Lv et al., 2020).

NTZ has been reported to modulate UPR signaling, promoting antioxidant defenses, suppressing inflammatory cytokines, inhibiting PDI, and ultimately preventing cell injury (Di Santo and Ehrisman, 2014; Di Santo and Ehrisman, 2013). The IRE1 induces mRNA splicing and induces “TNF-associated factor 2 (TRAF2),” prompting inflammation/apoptosis (Rajapaksa et al., 2015). Contrarily, NTZ induces glutathione S-transferase P1, which can mitigate TRAF2 (Müller et al., 2008). ATF6 induction activates ER chaperone protein release such as PDI (Fung et al., 2014), whereas NTZ plays a vital part in the maintenance of protective UPR response via PDI suppression (Di Santo and Ehrisman, 2014; Di Santo and Ehrisman, 2013). PERK induces phosphorylation for the eIF2α and promotes ATF4, thereby protecting the cell from oxidation (Sureda et al., 2020), while NTZ upregulates the ATF4 (Ashiru et al., 2014), demonstrating antioxidant activity. Furthermore, the activation of persistent UPR signaling and PDI is fundamental in the progression of liver disease (Maiers and Malhi, 2019; Mohan et al., 2019). To sum up, ER stress activates cytoprotective UPR that favors cell survival under stressful conditions. However, when these conditions remain unresolved, the UPR switches to proapoptotic and inflammatory pathways with persistent accumulation of misfolded proteins promoting cell death (Chen et al., 2023). Our findings demonstrated attenuated ER stress upon NTZ administration in MTX-challenged rats, which, at least in part, explains the depicted hepatoprotective effects of NTZ herein; this might be attributed to the observed alleviation of oxidative and inflammatory stressful conditions induced by MTX.

Indeed, NTZ and its active circulating metabolite tizoxanide directly protect from stress-induced apoptosis and necroptosis to alleviate liver damage in acute-on-chronic liver failure rat model (Bobowski-Gerard et al., 2024). Liu et al. (2024) demonstrated that nitazoxanide and tizoxanide activates AMPK, inhibits STAT3 and Smad2/3 activation, collagen I expression and secretion leading to inhibition of liver fibrosis induced by carbon tetrachloride-induced and bile duct ligation in mice (Liu et al., 2024). Further, nitazoxanide was reported to activate AMPK in HepG2 cells, mitigate high fat diet-induced hepatic steatosis in C57BL/6J mice and improve western diet-induced hepatic steatosis in *Apoe* ^−/−^ mice (Li et al., 2022); the authors recommended repurposing nitazoxanide for hepatic steatosis. These findings by other research groups support our findings which indicated that NTZ suppressed MTX-induced oxidative stress and ER stress and eventually the proapoptotic pathways protecting the liver against MTX toxicity.

In our attempt to scrutinize the hepatoprotective potential of NTZ and the possible implication of ER stress inhibition in our model of MTX-induced hepatotoxicity, we compared NTZ effects to a hepatoprotective NAC with documented ER stress inhibition and with a standard ER stress inhibitor (4-PBA) (Soliman et al., 2019). Both NAC and 4-PBA ameliorated MTX-induced hepatotoxicity as manifested by lowered serum liver function enzymes attenuated histopathological scores and restored normal histological features. NAC hepatoprotective effects may be attributed to its antioxidant capacity in drug-induced hepatotoxicity (Maheswari et al., 2014; Grisanti et al., 2018) and streptozotocin-diabetic rats (Erdogan et al., 2023). Similarly, 4-PBA strongly attenuated oxidative stress in a high-fat diet or ammonia nitrogen-induced liver injury related to its ER stress-inhibiting activity (Dong et al., 2022). The findings in the present work are congruent with Luo and colleagues’ report demonstrating the regulatory action of 4-PBA on ER stress-oxidative stress in diabetic nephropathy (Luo et al., 2010). NAC was reported to repress ER-stress-mediated apoptosis during liver ischemia/reperfusion injury, which aligns with our results (Sun et al., 2014). Several reports demonstrated the pronounced anti-inflammatory antioxidant effectiveness of NAC (Uraz et al., 2013; Tenório et al., 2021), which further supports our results. ER stress is known to be implicated in the pathology of many inflammatory disease models, including LPS-induced lung inflammation (Kim et al., 2013), high-fat diet-induced insulin resistance (Yuzefovych et al., 2013), or chronic graft-versus-host disease (Mukai et al., 2017). Suppression of ER stress with 4-PBA attenuated the severity of systemic inflammation in such inflammatory disorders and in our model of MTX-induced hepatic inflammation. NTZ, especially at the higher dose, elicited similar hepatoprotective effects comparable to NAC and 4-PBA, indicating the possible involvement of ER stress modulation in NTZ-elicited hepatoprotective effects.

## 5 Conclusion

Collectively, it can be concluded that MTX-induced hepatotoxicity is mediated, at least in part, through ER stress, which was confirmed by the reversal of MTX toxicity upon administration of 4-PBA and NAC. NTZ-elicited hepatoprotective effects are suggested to be mediated via modulation of PERK/CHOP10 signaling along with attenuated proapoptotic oxidative stress and proinflammatory signaling. NTZ replicated findings similar to those of the ER stress inhibitor (4-PBA) used in the study, further supporting our conclusion. NTZ is an FDA-approved drug with high safety and reasonable cost and has shown efficient hepatoprotective potential; hence, it can be recommended as adjuvant therapy alongside MTX to counteract its cytotoxic adverse effects.

This study has limitations, particularly the potential underdiagnosis of long-term MTX effects. Another limitation of this study is the lack of experiments exploring the molecular dynamics of NTZ in the improvement of MTX-mediated liver toxicity in more details in a cell culture model. However, because hepatotoxicity is a result of complex mechanisms, animal studies must be performed in association with such *in vitro* experiments. These factors should be taken into consideration in future studies.

## Data Availability

The raw data supporting the conclusions of this article will be made available by the authors, without undue reservation.
