# Correction: Nitazoxanide mitigates methotrexate hepatotoxicity in rats: role in inhibiting apoptosis and regulating endoplasmic reticulum stress

**DOI:** 10.3389/fphar.2026.1880862

**Published:** 2026-09-01

**Authors:** Nevertyty Mohamed Mahmoud, Shimaa M. Elshazly, Fatma El-shaarawy, Sawsan A. Zaitone, Afaf A. Aldahish, Gehan A. Ahmed, Manal S. Fawzy, Sheka Yagub Aloyouni, Sally Y. Abed, Tahani Saeedi, Shaimaa S. El-Sayed

**Affiliations:** 1 Department of Clinical Pharmacology, Faculty of Medicine, Zagazig University, Zagazig, Egypt; 2 Department of Pharmacology and Toxicology, Faculty of Pharmacy, Zagazig University, Zagazig, Egypt; 3 Department of Biochemistry, Faculty of Pharmacy, Sinai University, Arish, Egypt; 4 Department of Pharmacology and Toxicology, Faculty of Pharmacy, Suez Canal University, Ismailia, Egypt; 5 Department of Pharmacology and Toxicology, Faculty of Pharmacy, University of Tabuk, Tabuk, Saudi Arabia; 6 Department of Pharmacology, College of Pharmacy, King Khalid University, Abha, Saudi Arabia; 7 Forensic Medicine and Clinical Toxicology Department, Faculty of Medicine, Zagazig University, Zagazig, Egypt; 8 Department of Biochemistry, Faculty of Medicine, Northern Border University, Arar, Saudi Arabia; 9 Center for Health Research, Northern Border University, Arar, Saudi Arabia; 10 Research Department, Natural and Health Sciences Research Center, Princess Nourah bint Abdulrahman University, Riyadh, Saudi Arabia; 11 Department of Respiratory Care, College of Applied Medical Science in Jubail, Imam Abdulrahman Bin Faisal University, Jubail, Saudi Arabia; 12 Department of Pharmacology and Toxicology, School of Pharmacy, Taibah University, Medina, Saudi Arabia

**Keywords:** nitazoxanide, methotrexate, hepatotoxicity, endoplasmic reticulum stress, rat

There was a mistake in Table 1 as published. The accession number for GRP78, and the primer sequence, accession number and reference for PERK were incorrect. The corrected Table 1 appears below. The correct reference for PERK has been added to the reference list as Chen, D., Xu, T., Li, Y., Xu, J., Peng, B., Xu, W., et al. (2023). Stress regulation of WFS1 and PERK-p-eIF2α-ATF4 signaling pathway in placental tissue cells of intrahepatic cholestasis of pregnancy. Placenta 139, 1–11. doi: 10.1016/j.placenta.2023.05.018.

**TABLE 1:** The sequence of the primers used in the qRT-PCR experiment.

Target gene	Primer sequence: 5′–3′	Accession number	References
ATF4	F: TCCTCGATACCAGCAAATCC R: ACCCATGAGGTTTGAAGTGC	NM_024403.2	Soliman et al. (2019)
CHOP10	F: AGGTCCTGTCCTCAGATGAAA R: TAGGGATGCAGGGTCAAGAGT	NM_024134.2	Soliman et al. (2019)
GRP78	F:5′-GAC GCA CTT GGA ATG ACC CTT-3 R: 5′-TTGGTT TGC CCA CCT CCG AT-3′	NM_013083.2	Liu et al. (2018)
PERK	F: 5′- GCCACAGAGAACAGCGTGTA-3′ R: 5′-GATCGTCGGCAGAGGAATAA-3′	NM_031599.2	Chen et al. (2023)
β-actin	F: ATGGATGACGATATCGCTGC R: CTTCTGACCCATACCCACCA	NM_031144.3	Soliman et al. (2019)

There was a mistake in Figure 4B as published. The unit for Hepatic IL-1β on the y axis should be pg/mg tissue instead of pg/g tissue. The corrected Figure 4 appears below.

**FIGURE 4:**
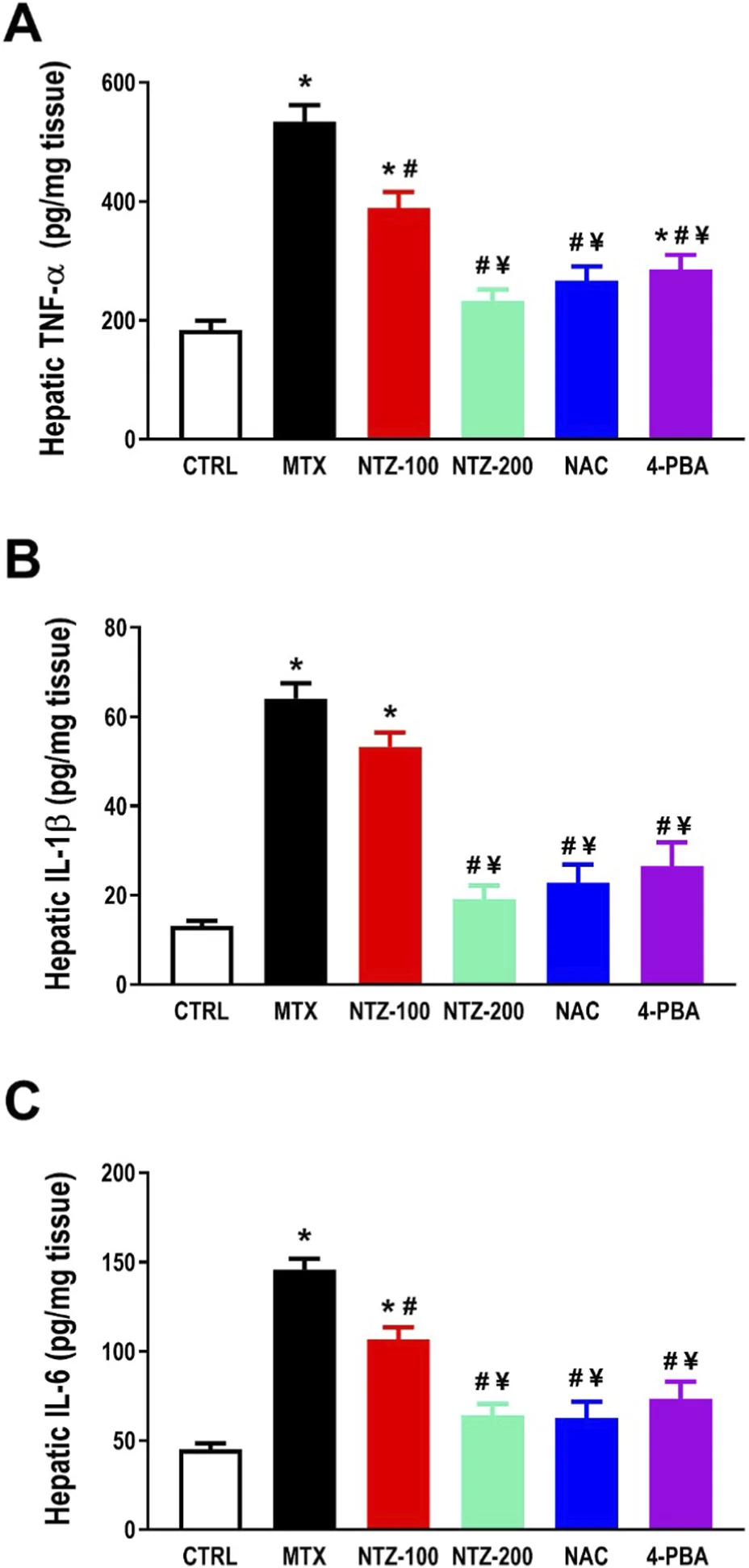
Effect of 10 days administration of nitazoxanide (NTZ-100 and NTZ-200), N-acetylcysteine (NAC, at 500 mg/kg), and 4-Phenylbutyric acid (4-PBA) on hepatic inflammation. Hepatic inflammation is demonstrated by increased hepatic levels of TNF-α, **(A)**, IL-1β, **(B)** and IL-6, **(C)**. Data presented here are mean ± SEM. * Vs. CTRL; # Vs. MTX group; ¥ Vs. NTZ-100 group, *p* < 0.05.

There was a mistake in Figure 6A as published. The unit for Hepatic IRE1 on the y axis should be ng/g tissue instead of ng/mg protein. The corrected Figure 6 appears below.

**FIGURE 6:**
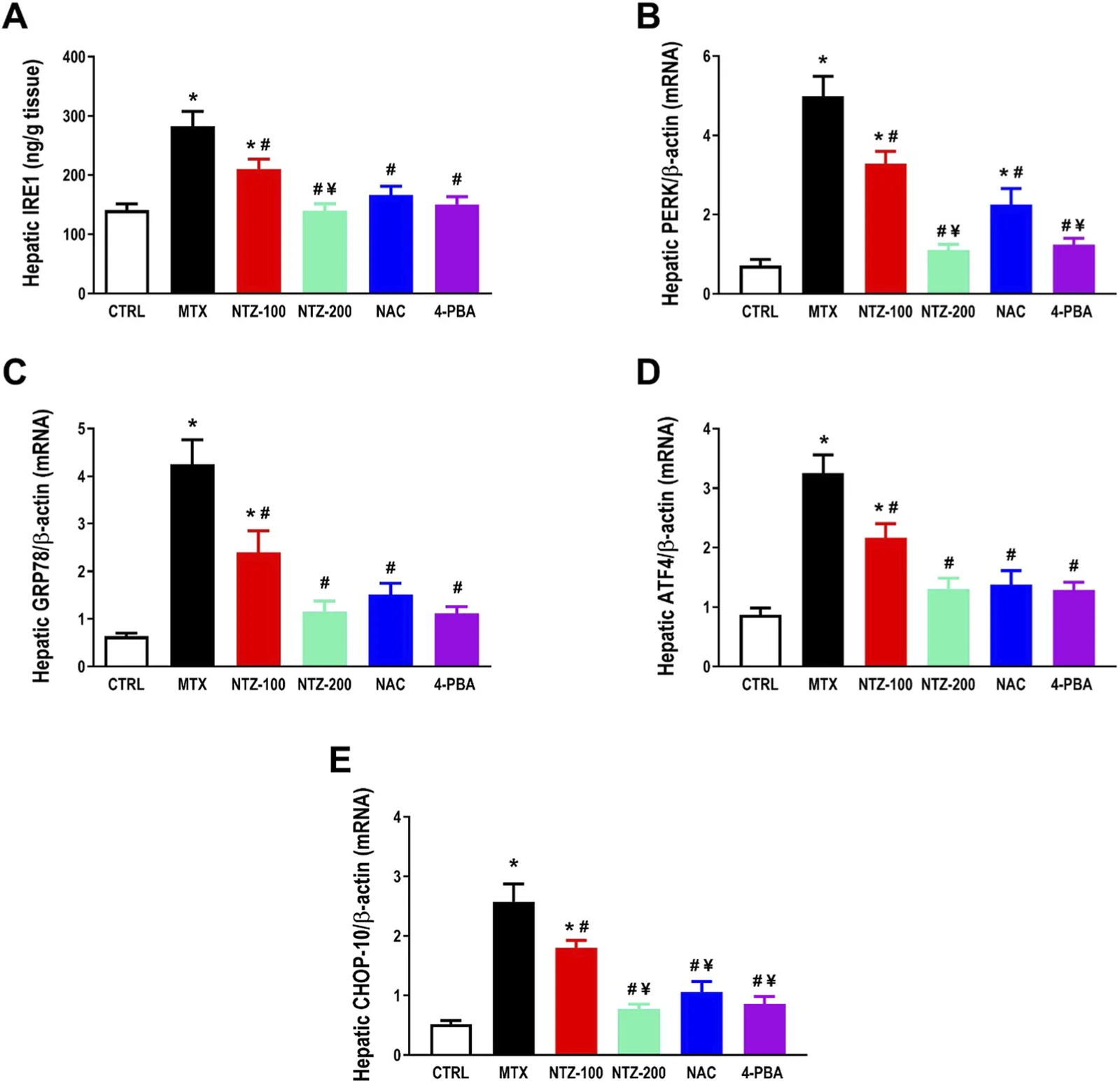
Effect of 10 days administration of nitazoxanide (NTZ-100 and NTZ-200) at 100 and 200 mg/kg, respectively, N-acetylcysteine (NAC) at 500 mg/kg and 4-Phenylbutyric acid (4-PBA) on hepatic endoplasmic reticulum (ER) stress. Hepatic ER-stress is expressed by hepatic levels of stress sensor protein, inositol-requiring enzyme 1 IRE1, **(A)**, and hepatic mRNA expression of PERK, **(B)**, GRP78, **(C)**, ATF4, **(D)**, and C/EBP homologous protein CHOP-10, **(E)**. Data are means ± SEM. * Vs. CTRL; # Vs. MTX group; ¥ Vs. NTZ-100 group, *p* < 0.05.

