# Supplementary material for: Nitazoxanide mitigates methotrexate hepatotoxicity in rats: role in inhibiting apoptosis and regulating endoplasmic reticulum stress
Source: Front Pharmacol. 2024 Dec 2;15:1491249. doi: 10.3389/fphar.2024.1491249 (PMC11647085; doi:10.3389/fphar.2024.1491249)
Supplement: Supplementary file 1 [file DataSheet1.docx]

Supplementary Material

# Supplementary Data

Permission is granted to Frontiers in Pharmacology to publish the following KEGG pathway map image in the article "Nitazoxanide mitigates Methotrexate hepatotoxicity in Rats: Role in inhibiting apoptosis and Regulating Endoplasmic Reticulum Stress" written by Sawsan A. Zaitone and colleagues:

- map04141 Protein processing in endoplasmic reticulum

subject to the condition that the original source is acknowledged by citing at least one KEGG paper.

Date: 5 August 2024
Copyright holder: Kanehisa Laboratories
